# Molecular mechanism by which CDCP1 promotes proneural-mesenchymal transformation in primary glioblastoma

**DOI:** 10.1186/s12935-021-02373-1

**Published:** 2022-04-11

**Authors:** Zhiying Lin, Zhu Zhang, Haojie Zheng, Haiyan Xu, Yajuan Wang, Chao Chen, Junlu Liu, Guozhong Yi, Zhiyong Li, Xiaoyan Wang, Guanglong Huang

**Affiliations:** 1 Jiangxi Provincial People’s Hospital, The First Affiliated Hospital of Nanchang Medical College, Nanchang, 330006 Jiangxi China; 2 Department of Neurosurgery, Nanfang Hospital, Southern Medical University, No. 1838 Guangzhou Avenue North, Guangzhou, 510515 Guangdong China; 3 The Laboratory for Precision Neurosurgery, Nanfang Hospital, Southern Medical University, Guangzhou, 510515 Guangdong China

**Keywords:** Glioma, CDCP1, Proneural-mesenchymal transition, Epithelial-mesenchymal transition, Prognostic risk model

## Abstract

**Background:**

Compared with the proneural (PN) subtype of glioblastoma (GBM), the mesenchymal (MES) subtype is more invasive and immune evasive and is closely related to poor prognosis. Here, we used transcriptome data and experimental evidence to indicate that CUB domain-containing protein 1 (CDCP1) is a novel regulator that facilitates the transformation of PN-GBM to MES-GBM.

**Methods:**

The mRNA expression data of CDCP1 in glioma were collected from the TCGA, CGGA and GEO databases, and in vitro experiments verified CDCP1 expression in glioma tissue samples. Independent prognostic analysis revealed the correlation of the CDCP1 expression level and patient survival. Bioinformatics analysis and experiments verified the biological function of CDCP1. Multivariate proportional hazards models and a PPI network were used to select key genes. A prognostic risk model for predicting the survival of glioma patients was constructed based on the selected genes.

**Results:**

The results showed that the expression of CDCP1 increased with increasing tumor grade and that the overexpression of CDCP1 correlated with a poor prognosis. CDCP1 was highly expressed in MES-GBM but weakly expressed in PN-GBM. The risk model (considering CDCP1 combined with CD44 and ITGAM expression) could represent a tool for predicting survival and prognosis in glioma patients.

**Conclusions:**

Our study indicates that CDCP1 plays an important role in facilitating the transformation of PN-GBM to MES-GBM.

**Supplementary Information:**

The online version contains supplementary material available at 10.1186/s12935-021-02373-1.

## Background

Glioma accounts for approximately 30% of all central nervous system (CNS) tumors and 80% of malignant primary brain tumors [1]. Despite the progress made in the past decade, glioblastoma (GBM, WHO grade IV) is still one of the most difficult tumor types to treat. The median survival time of glioblastoma patients is only 12–15 months [2]. According to the TCGA database, GBM has four intrinsic molecular subtypes: mesenchymal (MES), classical (CL), neural (NL), and proneural (PN) [3, 4]. The distinct molecular subtypes have prognostic value for predicting survival and can also be used to predict sensitivity to TMZ chemotherapy. Compared with those with the PN subtype, GBM patients with the MES subtype are more resistant to radiotherapy and chemotherapy and have increased invasiveness and a relatively poorer prognosis [5]. Previous studies have shown increased expression of immune response-related genes in MES-GBM [6–8]. Maria et al. found that MES-GBM was the most immunogenic among the four subtypes, while the proneural subtype was the least immunogenic [9].

With the development of sequencing technology, bioinformatics can be used to identify the key driving factors of each specific cancer patient, realize a more personalized cancer treatment plan, and pave the way for new drugs targeting specific proteins [10]. Tumor analyses based on The Cancer Genome Atlas (TCGA), Chinese Glioma Genome Atlas (CGGA), and Gene Expression Omnibus (GEO) databases have not only revealed a panorama of alteration signatures in the tumor-related genome but also established the basis for comparative studies of relevant types of tumors [11–13].

At present, immunotherapy is the most attractive therapy for glioma, and intensive research is underway [14]. Combined immunotherapy, such as the combination of PD-L1, indoximod (IDO), and CTLA-4 inhibitors, can encourage the immune system to recognize and attack tumor cells, thus improving the prognosis of patients [15, 16]. Lucio Palma showed that lymphocytic infiltration had a significant effect on the prognosis of GBM patients [17].

CUB domain-containing protein 1 (CDCP1) is a transmembrane glycoprotein that contains three extracellular CUB domains. In 2001, Scherl-Mostageer and coworkers first discovered its high expression in human colorectal and lung tumors [18]. Since then, an increasing number of studies have found that targeting CDCP1 is effective in preclinical models of lung [19, 20], prostate [21, 22], breast [23, 24], and ovarian [25, 26] cancers. CDCP1 plays a key role in the invasion, migration and drug resistance of various tumors [27–29]. Robin et al. revealed that patients with high expression of CDCP1 had poor prognosis [30]. Fei et al. indicated that the miR-1272/ADAM9/CDCP1 pathway may serve as a targetable pathway for the prevention of glioma [31]. Our previous studies suggested that the expression of CDCP1 in MES-GBM was significantly higher than that in PN-GBM [32], but the role and mechanism of CDCP1 in glioma are still unclear. Further work is needed to understand these molecular events.

In this study, data obtained from public datasets (TCGA, CGGA, and GEO) and specimens collected from resected glioma samples revealed that CDCP1 expression was higher in glioma tissue than in normal brain tissue. Moreover, high expression of CDCP1 correlated with a poor prognosis of glioma, as revealed by survival analysis. GO enrichment analysis, KEGG pathway analysis and experimental verification showed that CDCP1 was mainly involved in the Epithelial-mesenchymal transition (EMT) process and immune infiltration. Correlation (COR) analysis showed that CDCP1 was highly expressed in MES-GBM and weakly expressed in PN-GBM. CDCP1 was found to play an important role in facilitating the transformation from PN-GBM to MES-GBM (PMT). We established a risk model (which considered the expression of CDCP1 combined with CD44 and ITGAM) and verified that it can be used to predict prognosis in glioma/GBM.

## Materials and methods

### Clinical tissue sample collection

A total of 132 glioma tissue samples and 35 normal brain tissue samples were collected from the Department of Neurosurgery, Nanfang Hospital of Southern Medical University. The patients underwent surgery between 2016 and 2019 and did not receive chemotherapy or radiotherapy before surgery. A total of 132 glioma tissue samples (35 WHO II grade, 42 WHO III grade, and 55 WHO IV grade) were histologically and pathologically classified by pathologists according to the 2016 WHO standards. Thirty-five normal brain tissue samples (15 from women, 20 from men) were obtained from patients undergoing epilepsy surgery, and normal tissue samples around the tumor were obtained as controls. The Ethics Committee of Nanfang Hospital approved all experiments, and all patients signed written informed consent forms.

### Analysis of CDCP1 expression in various tumors in GEPIA

Gene Expression Profiling Interactive Analysis (GEPIA) is an online tool based on TCGA and Genotype-Tissue Expression (GTEx) data [33]. The expression of CDCP1 among various tumor patients and healthy people was assessed through the online GEPIA database.

### Patients and datasets

RNA-sequencing (RNA-seq) data and clinical information used in this study for bioinformatics analysis were obtained from public datasets, including GEO, TCGA (https://cancergenome.nih.gov/) and CGGA (http://www.cgga.org.cn/). We excluded patients whose overall survival (OS) data were not available. GSE50161 (https://www.ncbi.nlm.nih.gov/gds/?term=GSE50161) includes 13 normal samples and 34 GBM samples. The CGGA dataset contained 966 glioma samples (270 WHO II grade, 322 WHO III grade, and 374 WHO IV grade). The TCGA dataset contained 667 glioma samples (511 LGGs and 156 GBMs) and 5 normal samples.

### Independent prognostic analysis

Based on the median expression level of CDCP1 in glioma patients, the patients were divided into high expression groups and low expression groups. Then, survival analysis and an independent prognostic analysis were conducted with the "survival" package (p < 0.05). The correlations between CDCP1, IDH1, MGMT promoter methylation, and 1p/19q deletion were analyzed with the "ggpubr" package [34, 35].

### Functional analysis

Gene set enrichment analysis (GSEA) was employed to analyze the biological function of a single gene. To analyze the main function of the different genes, the "clusterProfiler" package [36] was used for GO and KEGG analyses. The p value cutoff was set as 0.05. The results were plotted by using the ggplot2 package. The results were annotated by Pathview in the R Bioconductor package (https://www.bioconductor.org/).

### Correlation analysis of different genes

With log_2_(fold change) > 0.5 and p < 0.05 as the screening criteria, the expression matrix was analyzed, and the differential genes related to CDCP1 were identified through the "pheatmap" and "limma" packages of the R language.

### Construction and module analysis of the PPI network

STRING is an online tool used to evaluate protein–protein interaction (PPI) networks [37]. The significantly differentially expressed genes were input into the STRING network, the confidence threshold was set as 0.15 [38], the PPI network of the differentially expressed genes was constructed, and the unconnected points were hidden. The PPI network obtained from STRING was introduced into Cytoscape software [39]. Cytoscape software was used to visualize the network. The MCODE plug-in of Cytoscape was used to identify the functional modules [40]. Submodules were sorted by score. The higher the score was, the stronger the protein correlation in the module.

### Centrality analysis of the PPI network and screening of key genes

The analysis of centrality determines the degree, betweenness, and closeness of network nodes [41]. Key genes were predicted by using the Cytoscape plug-in CytoNCA, and the degree centrality (DC), betweenness centrality (BC) and closeness centrality (CC) of the key genes were calculated. DC is a measure of the importance of a single node that describes the number of edges connecting nodes [42]. BC is the shortest path between any two nodes in the computing network [43]. CC is the average length of the shortest path from each node to other nodes [44, 45]. The top 2% of nodes for the three parameters were used for further analysis. Then, the top 2% of genes for each parameter were combined with the analysis results of the module, and the key genes with high centrality values were located in the first module.

### CIBERSORT

CIBERSORT was used to estimate the proportions of immune cells and stromal cells from normalized gene expression profiles with a deconvolution algorithm [46]. The immune cell subtypes included naive B cells, memory B cells, plasma cells, CD8 + T cells, naive CD4 + T cells, resting memory CD4 + T cells, activated memory CD4 + T cells, follicular helper T cells (Tfhs), regulatory T cells (Tregs), gamma delta T cells (γδ T cells), resting NK cells, activated NK cells, monocytes, M0 macrophages, M1 macrophages, M2 macrophages, activated dendritic cells, resting dendritic cells, activated mast cells, eosinophils, and neutrophils.

### ESTIMATE

The ESTIMATE algorithm in the estimate package of the R language was used to estimate the proportions of immune matrix components in the tumor microenvironment (TME) of each sample, and the results were presented in the form of three scores, namely, the immune score, stromal score and ESTIMATE score, which are positively correlated with immunity, the matrix and their sum. Therefore, the higher the score, the greater the proportions of corresponding components in the TME.

### Construction of a prognostic risk model

A prognostic risk model was constructed to evaluate the accuracy of the prognostic models with a single variable, and a multivariate prognostic model was constructed based on the area under the curve (AUC) of the receiver operating characteristic (ROC) curve. The prognostic risk models comprising a single gene and multiple genes were constructed by using the "pROC" package of the R language. The multivariate analysis was based on the results of the univariate analysis. ROC curves show the sensitivity and specificity of a binary diagnostic decision for varying cutoff points based on a single quantitative diagnostic variable or based on multiple diagnostic variables.

### Construction of the prognostic risk model

A multivariate Cox regression model (including patient age, sex, and WHO grade) was used to evaluate the relationship between each gene and the OS of glioma patients with the R programming language. P < 0.05 was considered statistically significant. Risk characteristics were established according to the regression coefficient of weighted gene expression, and the risk score formula was constructed as follows:

$$risk\,model = \sum\limits_{{n = 1}}^{I} {\left( {{\text{Exp}}_{n} *HR_{n} } \right)}$$

In the formula, I is the number of selected genes, Exp_n_ is the expression value of each gene, and HR_n_ is the multivariate Cox regression hazard ratio (HR). Glioma patients were divided into low-risk and high-risk groups according to the median risk score, and the performance of prognostic risk characteristics was measured by Kaplan–Meier analysis. The results were visualized as survival curves by the R package "survival". To better predict the 1-year, 3-year, and 5-year survival rates of glioma patients, the risk signature and several clinicopathological factors were included, and a nomogram was established by using the "rms" package of R based on the results of the multivariate analysis.

### Western blot analysis

Western blotting was performed according to our previous studies [47] with rabbit polyclonal antibodies against CDCP1 (Cell Signaling Technology, catalog 4115S, human, 1:1000), N-cadherin (Cell Signaling Technology, catalog #13116, human, 1:1000), vimentin (Cell Signaling Technology, catalog #5741, human, 1:1000), slug (Cell Signaling Technology, catalog #9585, human, 1:1000), and CD44 (Abcam, catalog #ab189524, human, 1:1000). An HRP-conjugated anti-rabbit or anti-mouse IgG antibody was used as the secondary antibody (Cell Signaling Technology, catalog #5174S, human, 1:2000). Signals were detected using enhanced chemiluminescence reagents (Pierce, Rockford, IL, USA).

### Immunohistochemistry

Paraffin sections were deparaffinized and rehydrated. Heat-induced antigen retrieval was carried out for 15 min in citrate buffer. After endogenous peroxidase was blocked with 3% hydrogen peroxide and nonspecific antigens were blocked with 5% bovine serum albumin, incubation was performed with antibodies against CDCP1 (Abcam, catalog #ab1377, human, 1:100), CD44 (Abcam, catalog #ab189524, human, 1:100), and ITGAM (Cell Signaling Technology, catalog #23743, human, 1:100). The next day, the secondary antibody was added after washing with PBS three times. Subsequently, sections were counterstained with hematoxylin before examination by microscopy.

### Immunohistochemistry staining evaluation

Two pathologists examined and scored the immunohistochemically stained sections without knowledge of the clinical parameters. Staining intensity was scored as 0 (negative), 1 (weak), 2 (moderate), or 3 (strong). The positive staining area was classified with a score of 0 (< 5%), 1 (6–25%), 2 (26–50%), 3 (51–75%), and 4 (> 76%).

### Cell culture and lentivirus infection

The human glioma cell lines U87 and LN229 and the human colorectal carcinoma cell line HCT116 were purchased from the American Type Culture Collection (ATCC). In the laboratory, all cell lines were grown in Dulbecco’s modified Eagle’s medium (DMEM) (Biological Industries) supplemented with 10% fetal bovine serum (FBS, Gemini Foundation). We used a lentivirus (LV) encoding green fluorescent protein (eGFP, 30 kDa) and an LV encoding CDCP1 cDNA (LV‑CDCP1, Lot# EX-H2069-Lv122, GeneCopoeia) of the eGFP gene. Then, LV-CDCP1 and the lentivirus of the negative control group carrying eGFP (LVCon, GeneCopoeia) were used to infect U87 and LN229 glioma cells. Further analysis was performed 72 h post transfection.

### Cell migration assay

Cell migration assays were carried out with Transwell assays. Approximately 5 × 10^4^ cells in 100 μL DMEM were seeded onto a polycarbonate membrane inserted into a Transwell chamber (BD Biosciences). Five hundred microliters of complete medium was added as a chemoattractant in the lower chamber. After the cells were incubated for the appropriate time, the adherent lower chamber cells were fixed with paraformaldehyde and stained with 0.2% crystal violet solution. The images were captured in five predetermined fields under a microscope.

### Statistical analysis

The R language (version 3.5.3) was used for statistical analysis. Kaplan–Meier survival curves based on each key gene, forest maps of the independent prognostic variables of each key gene, and box diagrams of multiple variables were generated. P < 0.05 (bilateral) was considered statistically significant.

## Results

### CDCP1 mRNA expression increases with increasing glioma grade

**Fig. 1:**
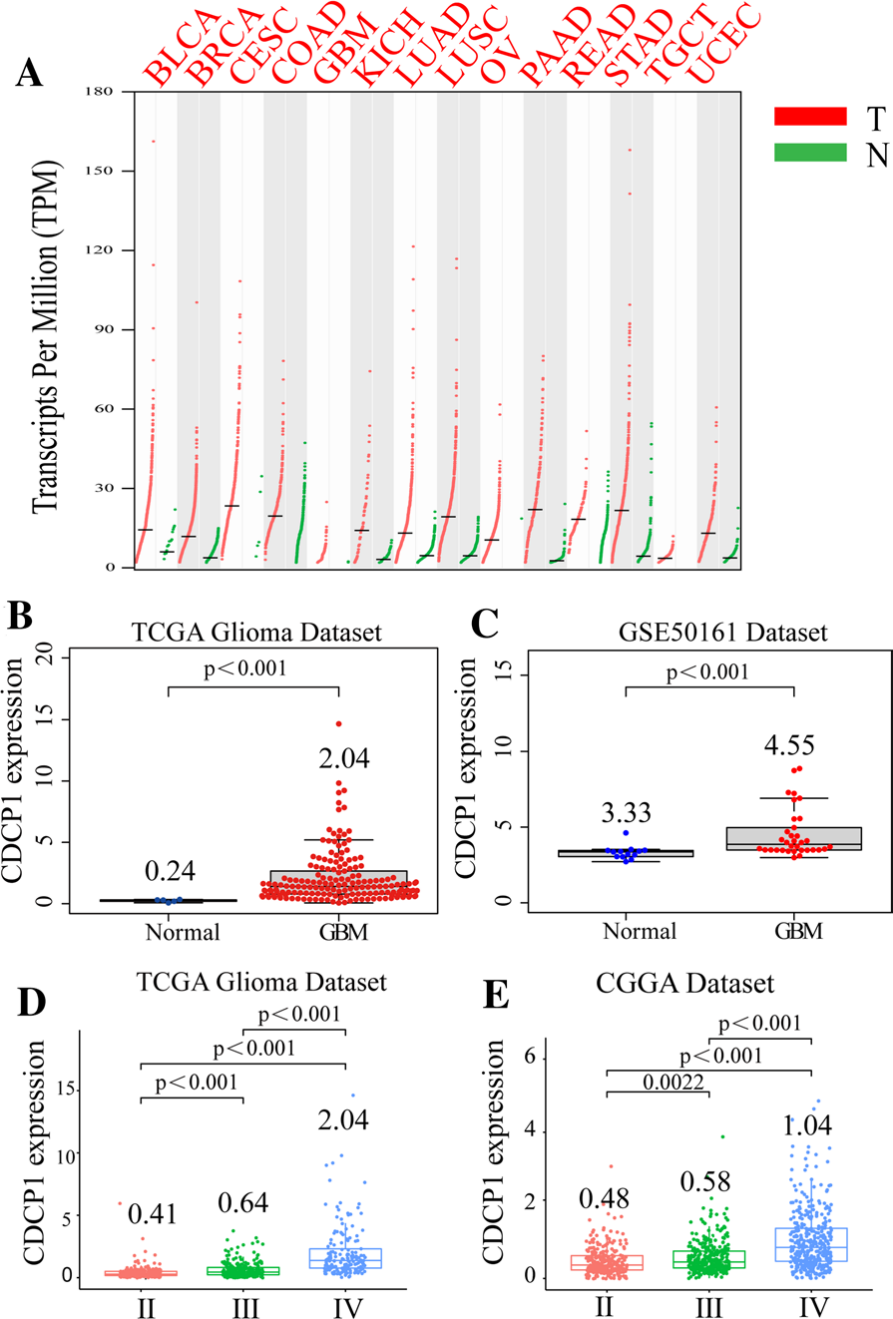
The expression of CDCP1 in glioma. **A** The expression of CDCP1 in GBM and other tumors (*BLCA* bladder urothelial carcinoma, BRCA: breast invasive carcinoma, *CESC* cervical squamous cell carcinoma and endocervical adenocarcinoma, *COAD* colon adenocarcinoma, *GBM* glioblastoma, *KICH* kidney chromophobe, *LUAD* lung adenocarcinoma, *LUSC* lung squamous cell carcinoma, *OV* ovarian serous cystadenocarcinoma, *PAAD* pancreatic adenocarcinoma, *READ* rectum adenocarcinoma, *STAD* stomach adenocarcinoma, *TGCT* testicular germ cell tumors, *UCEC* uterine corpus endometrial carcinoma). In the TCGA data **B** and GSE50161 (**C**), the expression of CDCP1 in GBM tissue was significantly higher than that in normal brain tissue. In the TCGA data **D** and CGGA data (**E**), CDCP1 mRNA expression increased with increasing glioma grade

CDCP1 has been widely studied in various tumors, and the expression of CDCP1 in various common tumors was analyzed through GEPIA. The expression of CDCP1 in bladder urothelial carcinoma (BLCA), breast invasive carcinoma (BRCA), cervical squamous cell carcinoma and endocervical adenocarcinoma (CESC), colon adenocarcinoma (COAD), glioblastoma (GBM), kidney chromophobe (KICH), lung adenocarcinoma (LUAD), lung squamous cell carcinoma (LUSC), ovarian serous cystadenocarcinoma (OV), pancreatic adenocarcinoma (PAAD), testicular germ cell tumors (TGCTs) and uterine corpus endometrial carcinoma (UCEC) was significantly increased (Fig. 1A).

**Fig. 2:**
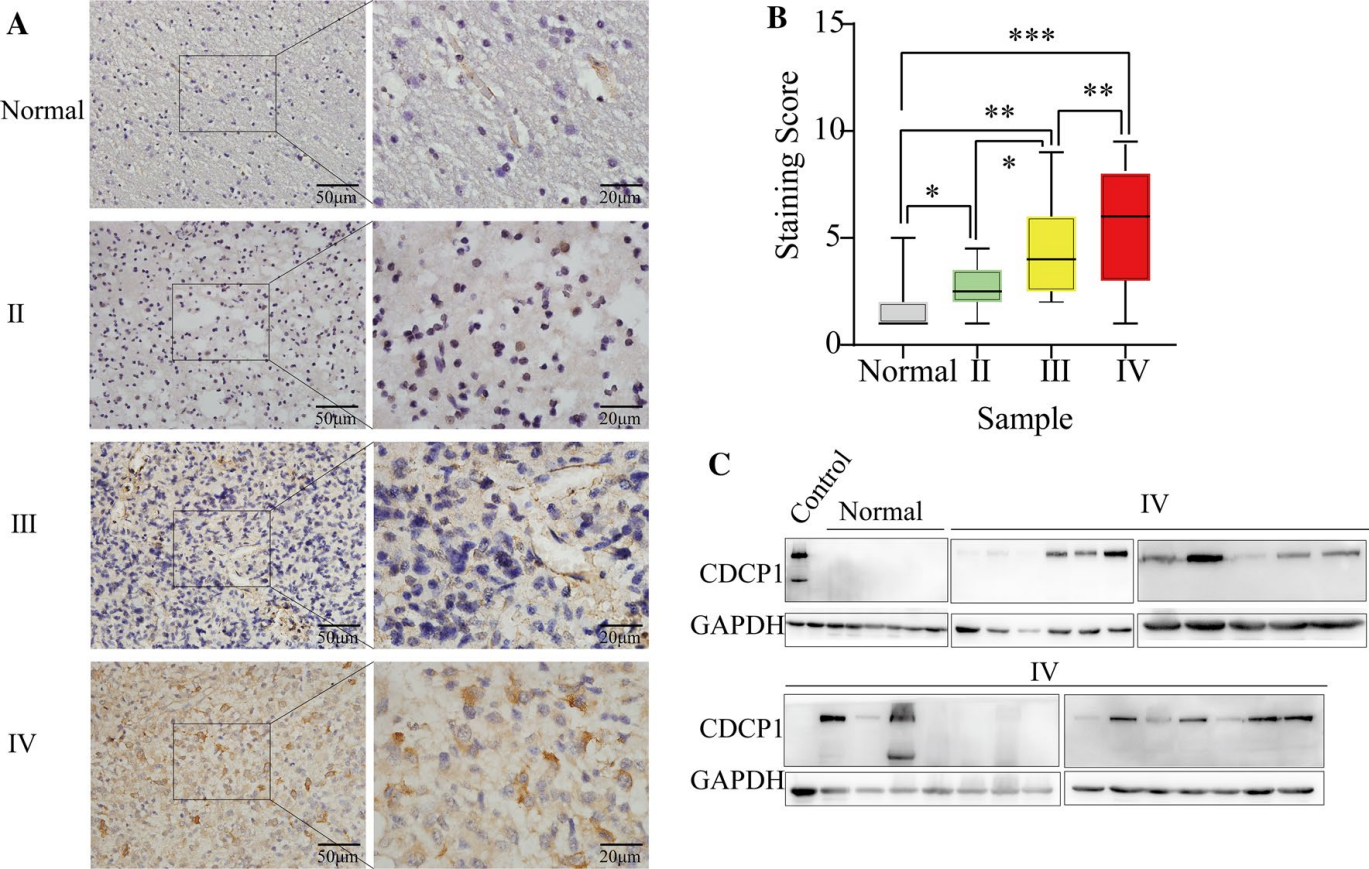
The expression of CDCP1 was verified in glioma specimens from a southern hospital. Immunohistochemistry **A** verified CDCP1 expression in 35 samples of WHO grade II, 42 samples of WHO grade III and 55 samples of WHO grade IV glioma, and analysis of the immune scores **B** demonstrated that CDCP1 expression increased significantly with increasing WHO grade. Western blot **C** confirmed that CDCP1 expression was significantly increased in GBM (in the HCT116 human colorectal cancer cell line with high CDCP1 expression)

To clarify the role of CDCP1 in human glioma, CDCP1 mRNA expression was measured in 698 glioma tissues and 5 normal tissues from the TCGA database, 970 glioma tissues from the CGGA database, and 34 GBM tissues and 13 normal tissues from the GSE50161 dataset. According to the analysis of the TCGA and GEO databases, CDCP1 expression was higher in GBM tissues than in normal brain (NB) tissues (Fig. 1B, C  p< 0.0001). CDCP1 mRNA significantly increased with increasing WHO grade (Fig. 1D, E). To further confirm these results, we examined the CDCP1 expression level in glioma tissues (grade II, n = 35; grade III, n = 42; and grade IV, n = 55) (Additional file 1: Table S10) and normal brain tissues (n = 35) by immunohistochemistry (Fig. 2A, B). Consistent with the results described above, CDCP1 expression significantly increased with increasing WHO grade. Furthermore, we examined the protein expression of CDCP1 in 27 GBM tissues and 6 normal brain tissues and found that CDCP1 was highly expressed in GBM (Fig. 2C).

### Patients with high CDCP1 expression have a poor prognosis

**Fig. 3:**
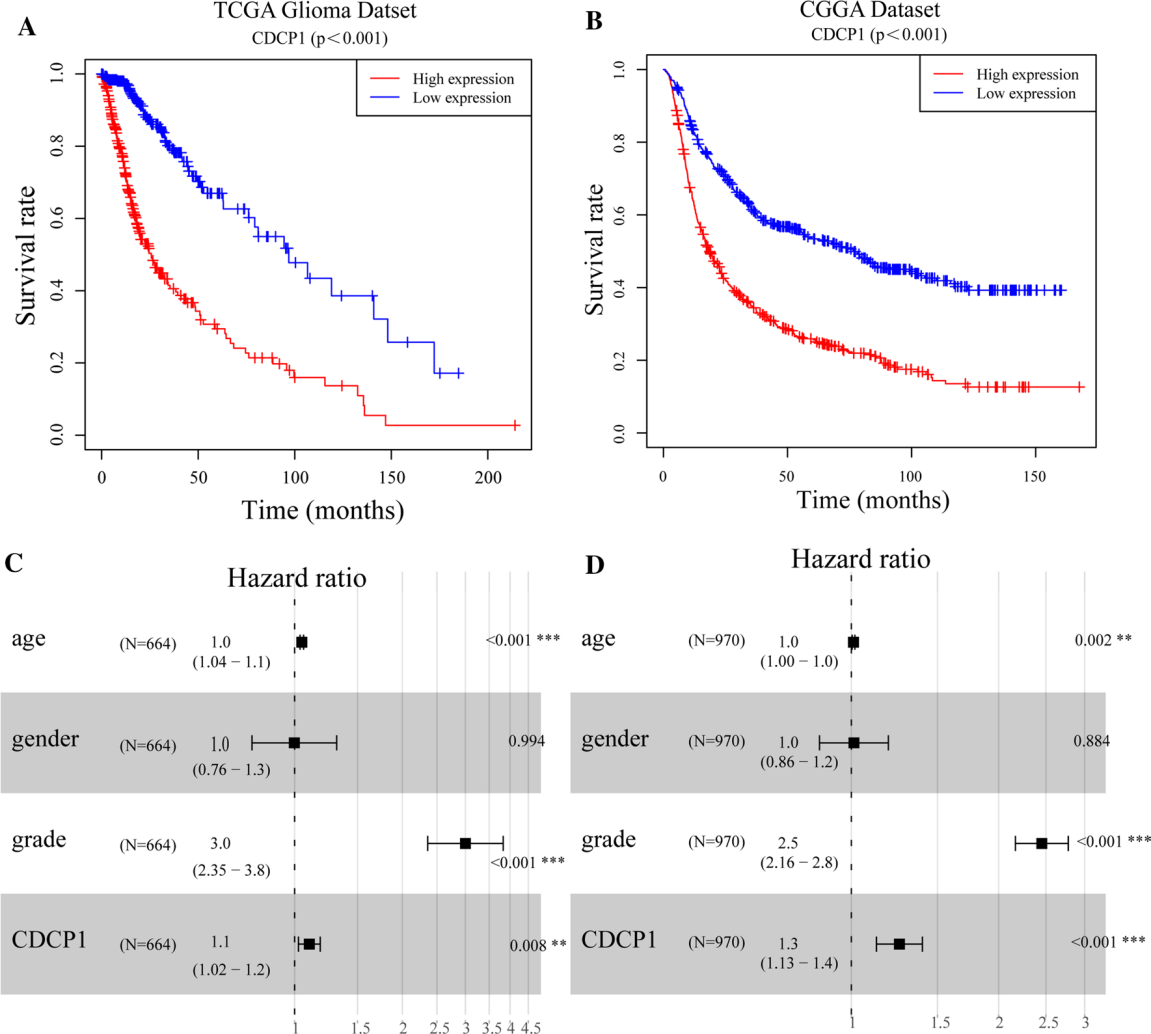
Kaplan–Meier survival curve and multivariate Cox regression analyses of the CDCP1 risk score in the TCGA **A**–**C** (n = 667) and CGGA (B-D) datasets(n = 966)

To investigate the prognostic value of CDCP1 expression in glioma, Kaplan–Meier analysis with the log-rank test was used to examine the relationship between the expression of CDCP1 and patient survival. With survival data obtained from the TCGA and CGGA databases, we assessed whether higher CDCP1 expression was associated with worse overall survival (OS). The median OS times of glioma patients with high and low expression of CDCP1 were 13 and 20 months, respectively, in the TCGA database (Fig. 3A, p< 0.0001). The median OS times of glioma patients with high and low expression of CDCP1 were 18 and 41 months, respectively, in the CGGA database (Fig. 3B,  p< 0.0001). Then, multivariate Cox regression analysis was performed to determine the prognostic value of CDCP1, and the results showed that CDCP1 was an independent prognostic factor in TCGA and CGGA data. Independent prognostic analysis showed that the HR was 1.1 (95% confidence interval, CI: 1.02–1.2) in the TCGA data and 1.3 (95% confidence interval, CI: 1.13–1.4) in the CGGA data (Fig. 3C, D). In the CGGA database analysis, we also found that CDCP1 was expressed at higher levels in the unmethylated MGMT promoter CPG island group than in the methylated MGMT promoter CPG island group (p < 0.0001) (Additional file 1: Fig. S1A). The expression of CDCP1 in the 1p/19q codeletion group was higher than that in the group without 1p/19q deletion (Additional file 1: Fig. S1B). The expression of CDCP1 in the IDH1 wild-type (WT) group was higher than that in the IDH1 mutant (MUT) group (Additional file 1: Fig. S1C).

### The expression of CDCP1 is positively correlated with invasion, migration and immune infiltration

**Fig. 4:**
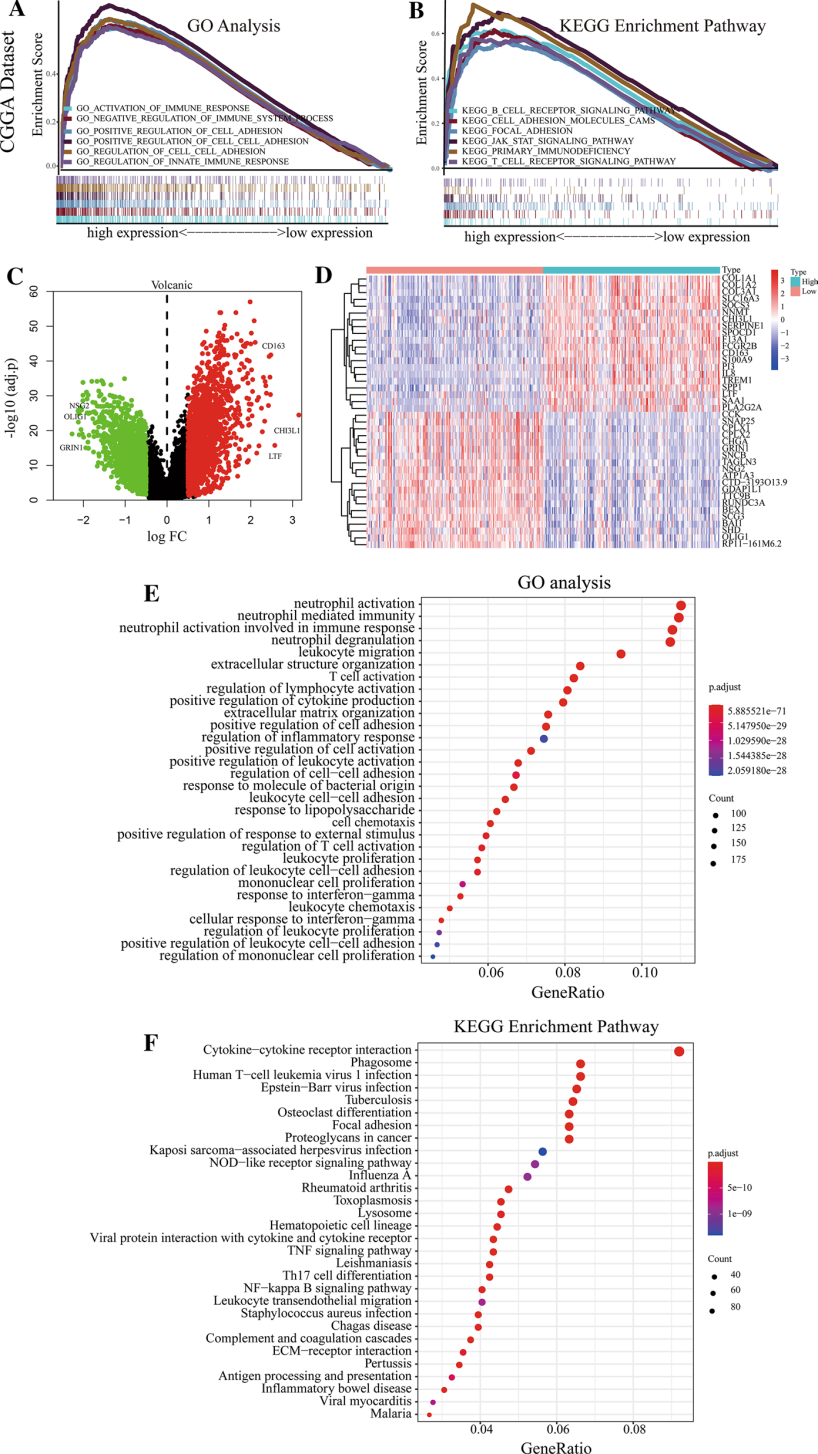
Functional analysis of CDCP1 in GBM. GSEA (A-B) of GO functions and KEGG pathways of CDCP1 in the CGGA data. Identification of differentially expressed genes related to changes in CDCP1 expression (**C**–**D**). GO functional analysis **E** and KEGG pathway analysis **F** of 2408 upregulated genes

We analyzed the molecular mechanism by which CDCP1 promotes the malignant progression of GBM, and gene set enrichment analysis (GSEA) was used to predict the possible biological functions of CDCP1 in GBM. TCGA data and CGGA data showed that the expression of CDCP1 mRNA was significantly correlated with EMT-related processes (such as cell adhesion, focal adhesion, and cell migration) and immune infiltration processes (such as lymphocyte migration, interleukin 6 production, and B cell receptor signaling) (Fig. 4A, B and Additional file 2: Fig. S2A, B).

To analyze the molecular mechanism by which CDCP1 promotes the malignant progression of GBM, we further mined the gene expression matrix of the CGGA database. After data processing and data analysis of the gene expression matrix of the CGGA database, 970 samples were analyzed. We screened 4007 related differentially expressed genes (1923 downregulated, 2084 upregulated) (Fig. 4C). The 20 upregulated genes and 20 downregulated genes most related to CDCP1 expression were visualized in the form of a heatmap (Fig. 4D). We found that the 40 genes with the strongest correlation with CDCP1 were also significantly correlated (Additional file 3: Fig. S3); for example, COL1A1 was negatively correlated with Oligo1 and positively correlated with COL1A2. GO functional analysis and KEGG pathway enrichment analyses were carried out for the 1923 downregulated genes and 2084 upregulated genes. The 2084 upregulated genes were associated with immune infiltration-related functions and EMT process-related functions. For example, the GO and KEGG terms related to immune infiltration were response to regulation of T cell activation, regulation of the immunological response, leukocyte migration, and leukocyte migration. The GO and KEGG terms related to the EMT process were response to regulation of cell–cell adhesion, positive regulation of cell adhesion, and the NF-κB signaling pathway (Fig. 4E, F). The possible biological functions of the 1923 downregulated genes were analyzed, and the cAMP signaling pathway and oxidative phosphorylation were mainly enriched (Additional file 4: Fig. S4A, B).

### Overexpression of CDCP1 promotes GBM cell migration

**Fig. 5:**
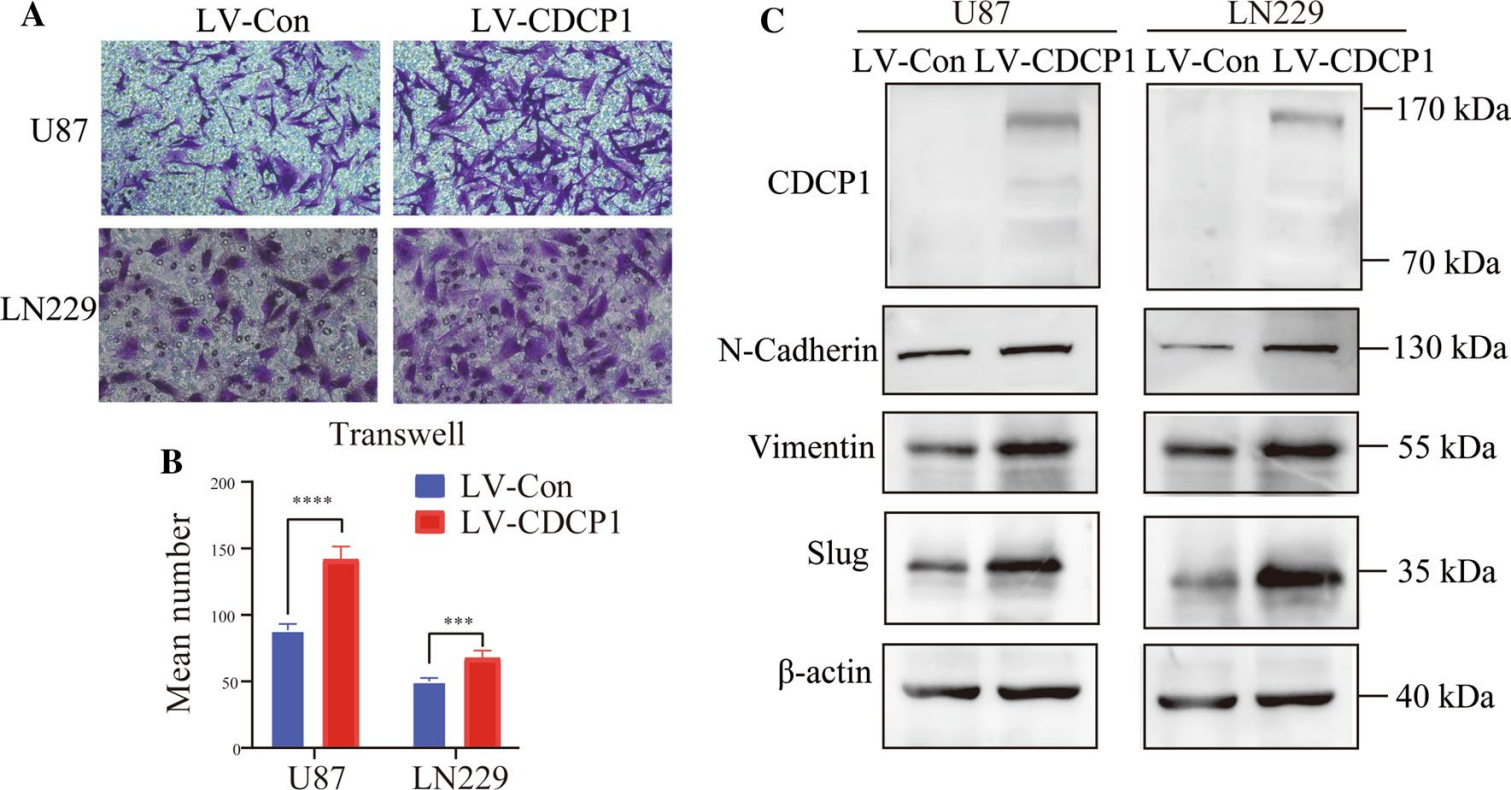
Verification of CDCP1 function in vitro. Transwell assays **A** and cell migration assays **B** showed that CDCP1 overexpression promoted the migration ability of glioma cells in vitro ( Wilcoxon rank-sum test)(the magnification is 200 times). Western blotting **C** was performed to detect the expression of N-cadherin, vimentin and Slug in the LV-CDCP1 and LV-Con groups

To confirm the biological function of CDCP1 in GBM revealed by the bioinformatic analysis, the expression of several EMT-associated proteins was examined in U87 and LN229 cells. After CDCP1 overexpression in U87 and LN229 cells, the expression of N-cadherin, vimentin, and slug was upregulated (Fig. 5A). In the Transwell assay, the percentage of migrated cells in the LV-CDCP1 group was significantly higher than that in the LVCon group (p < 0.05) (Fig. 5B, C).

### Identification of key genes

**Fig. 6:**
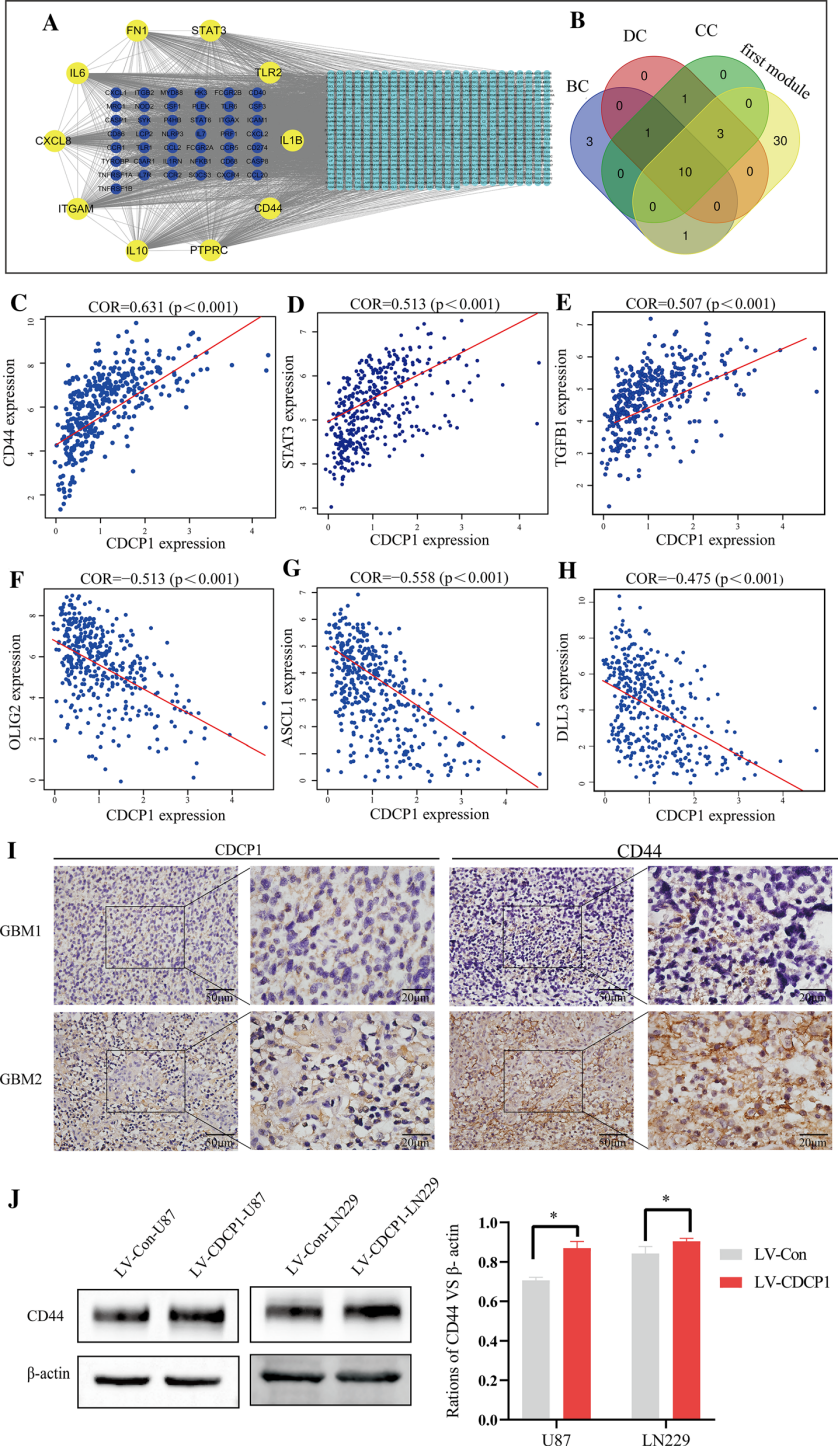
Analysis of the molecular mechanism of CDCP1 in GBM. Identification of key genes in the PPI network (A-B). High expression of CDCP1 was associated with MES-GBM. The correlation between CDCP1 expression and the mesenchymal **C**–**E** or proneural **C**–**H** signature was assessed using the CGGA database. Immunohistochemistry verification of the correlation between CDCP1 and CD44 expression (**I**). Western blot verification of the correlation between CDCP1 and CD44 expression **J** (Wilcoxon rank-sum test)

Analysis of the correlation between the 4007 related differentially expressed genes and CDCP1 yielded 789 highly correlated differentially expressed genes (the criteria were correlation coefficient = 0.5 and p < 0.05). To further explore the possible specific molecular mechanism by which CDCP1 affects the prognosis of GBM patients from a systematic perspective, a PPI network was constructed through the online STRING database. Then, the PPI network was imported into Cytoscape. The PPI network consisted of 718 genes and 8097 edges. In the PPI network, the top 5 genes with the greatest weight were IL6 (degree = 216), ITGAM (degree = 165), PTPRC (degree = 164), IL10 (degree = 160), and CXCL8 (degree = 158) (Fig. 6A).

To identify more closely related key genes in the complex PPI network, we used MCODE to conduct a module analysis of the network. We found 25 modules in the PPI network. The first module, composed of 44 genes that had the strongest interaction, scored 36.61. This module was located at the center of the entire network and included 44 nodes and 787 edges. The results indicated that the protein associations in the first module may be the strongest and most important part of the entire network.

To further assess the key genes in the complex PPI network, centrality analysis was performed. We studied the top 2% of the related genes of each parameter and obtained the degree, betweenness, and closeness of 11 genes by taking the intersection. Combined with the results of the module analysis, STAT3, PTPRC, FN1, IL1B, CXCL8, CD44, TLR2, IL10, IL6, and ITGAM were identified for further analysis because these ten genes with high centrality values were located in the first module. Among the key genes associated with CDCP1, CD44 and STAT3 are markers of MES-GBM (Fig. 6B). Therefore, we suspect a potential connection between CDCP1 and MES-GBM.

### Overexpression of CDCP1 promotes the transformation of PN-GBM to MES-GBM

In the past decade, according to the molecular phenotype, transcriptome and methylation analyses have classified GBM tumors into four subtypes: the anterior nerve type (PN-GBM), nerve type (NL-GBM), classical type (CL-GBM) and interstitial type (MES-GBM). According to these four types of GBM, we found through a database analysis that CDCP1 was highly expressed in MES-GBM and weakly expressed in PN-GBM (Additional file 1: Fig. S5).

The expression of CDCP1 was positively correlated with the expression of mesenchymal (MES) GBM markers such as CD44, STAT3, and TGFB1, while the expression of CDCP1 was negatively correlated with the expression of Olig2, ASCL1 and DLL3 in proneural (PN) GBM (Fig. 6C–H). The high expression of a combination of mesenchymal markers (e.g., CD44 and STAT3) is reminiscent of the EMT process that has been linked to dedifferentiated and transdifferentiated tumors [48]. The increase in CDCP1 expression may promote the transformation of PN-GBM to MES-GBM, which is associated with poor prognosis. Immunohistochemistry of 55 GBM patients confirmed that CDCP1 was significantly positively correlated with CD44 protein expression (Fig. 6I), with a correlation coefficient of 0.531 (Table 1). In U87 and LN229 cells, compared with the LVCon group, the LV-CDCP1 group had upregulated CD44 expression (Fig. 6J). In conclusion, these data suggest that CDCP1 is overexpressed in MES-GBM and prove that the overexpression of CDCP1 is significantly correlated with increased invasiveness and a relatively poor prognosis.

**Table 1:** Correlation between the protein expression of CDCP1 and CD44 in GBM tissue samples

CD44	CDCP1(Number of case)		COR	P
	Positive	Negative		
Positive	29	5	0.531	< 0.01
Negative	7	14		

### Verification of the significant positive correlation between CDCP1 expression and immune infiltration

We analyzed GBM data from the CGGA database and obtained the proportions of 22 common tumor-infiltrating immune cells in each sample with the CIBERSORT method. The tumor-infiltrating immune cells in GBM patients were mainly M2 macrophages (Additional file 6: Fig. S6A). Next, the correlations between these 22 kinds of tumor-infiltrating immune cells was analyzed. The results showed that memory B cells were negatively correlated with naive B cells, and CD8^+^ T cells were positively correlated with activated memory CD4^+^ T cells (Additional file 6: Fig. S6B).

As shown in Additional file 7: Fig. S7, with an increase in the expression of CDCP1, the expression levels of CD44, STAT3, TGFB1, CXCL8, FN1, IL1B, IL10, IL6, and ITGAM increased, whereas the expression levels of OLIG2, ASCL1, DLL3, BEX1, CDK5R1, CKB, NRXN2, CSPG5 and MAP2 decreased. The results also verified that the expression of CDCP1 was negatively correlated with the expression of PN-GBM markers (BEX1, CDK5R1, CKB, NRXN2, CSPG5 and MAP2).

**Fig. 7:**
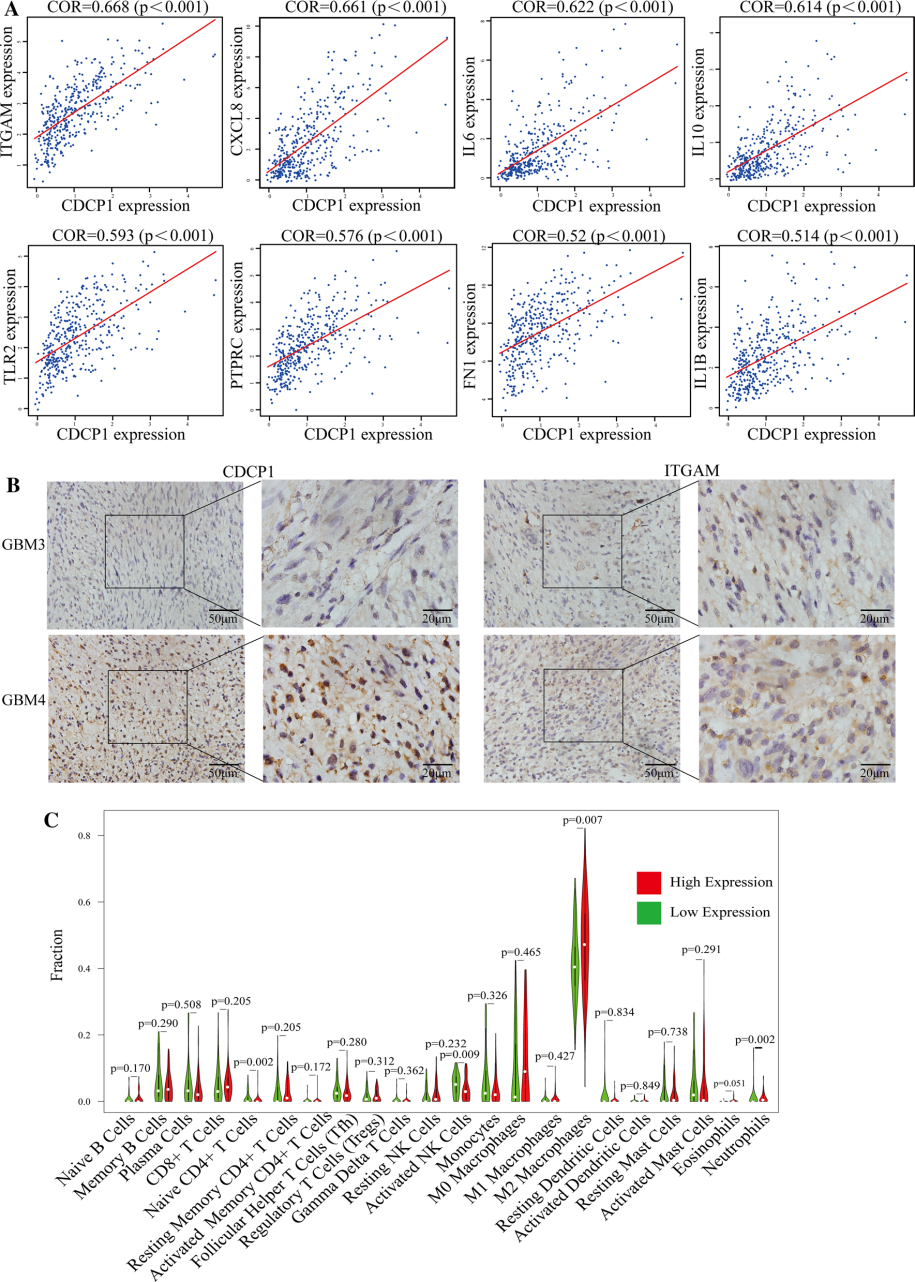
Analysis of CDCP1 in immune infiltration in GBM. CDCP1 was positively correlated with immune infiltration markers (**A**). Immunohistochemistry verification of the correlation between CDCP1 and ITGAM expression (**B**). Correlation analysis between CDCP1 expression and various types of infiltrating immune cells (**C**)

As shown in Fig. 7A, the expression of CDCP1 was positively correlated with the expression of immune infiltration markers (PTPRC, FN1, IL1B, CXCL8, CD44, TLR2, IL10, IL6 and ITGAM), with correlation coefficients above 0.5. Immunohistochemistry analysis of 55 samples from GBM patients confirmed that CDCP1 was significantly positively correlated with ITGAM protein expression, with a correlation coefficient of 0.565 (Fig. 7B and Table 2). With CIBERSORT analysis, we found that the expression of CDCP1 was related to a variety of infiltrating immune cells. The tumor tissues with high CDCP1 expression showed a specific immunophenotype, with prominent M2 macrophages (P = 0.007). There were significant differences in the proportions of CD4^+^ T cells, activated NK cells and neutrophils between tumors with high and low CDCP1 expression (p < 0.05) (Fig. 7C). Through the ESTIMATE algorithm, we found that the stromal score, immune score and ESTIMATE score in the GBM group with high CDCP1 expression were significantly higher than those in the GBM group with low CDCP1 expression (Additional file 7: Fig. S7A–C).

**Table 2:** Correlation between the protein expression of CDCP1 and ITGAM in GBM tissue samples

ITGAM	CDCP1(Number of case)		COR	P
	Positive	Negative		
Positive	27	3	0.565	< 0.01
Negative	9	16		

### The prognostic risk model based on CDCP1, CD44 and ITGAM has high diagnostic value

**Fig. 8:**
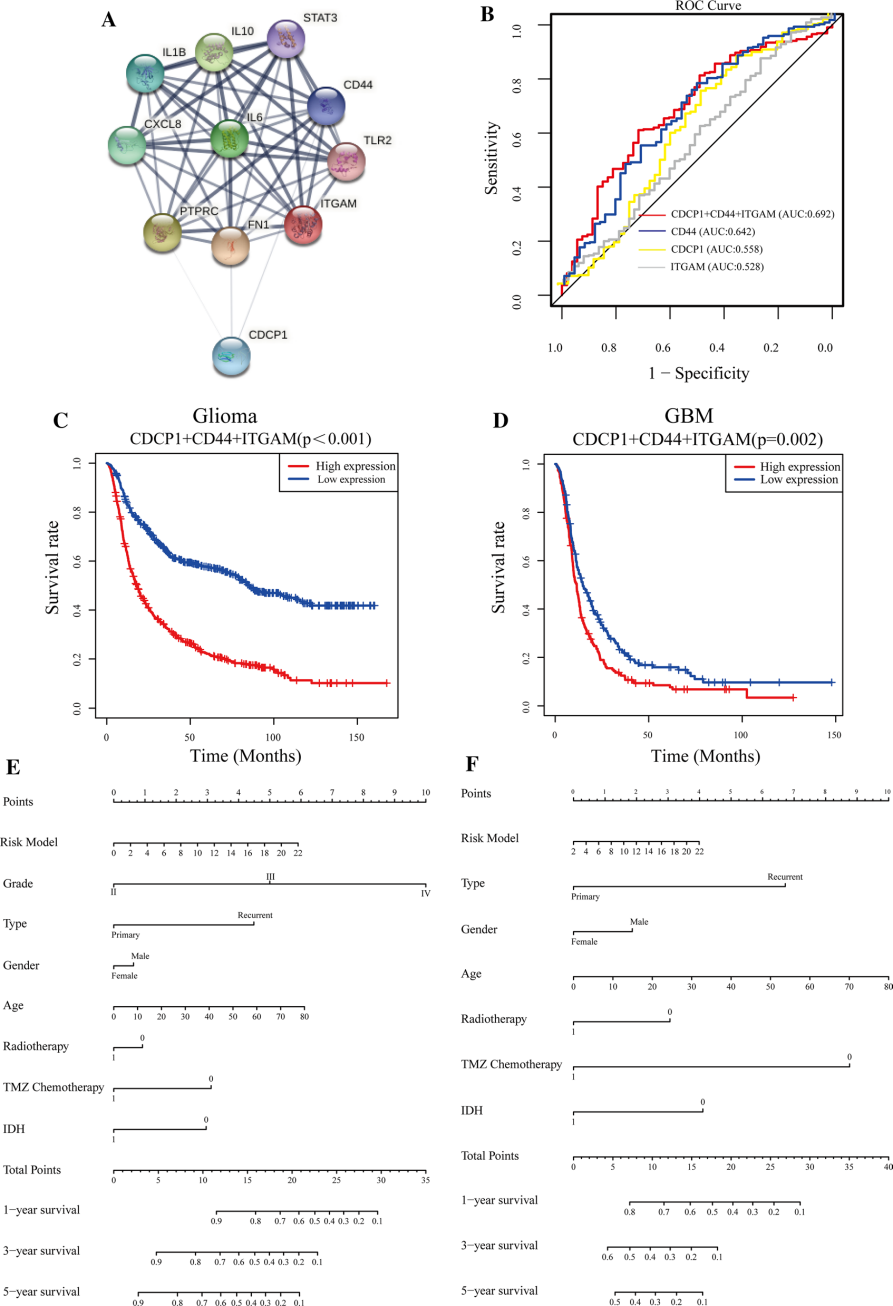
Establishment and verification of the prognostic risk model. Construction of a PPI network with key genes and CDCP1 (**A**). Establishment of the prognostic risk model comprising CDCP1, CD44 and ITGAM expression (**B**). Survival curves for glioma **C** and GBM **D** patients based on the prognostic risk model. Nomogram based on the risk model and clinicopathological factors. E: Glioma patient data. F: GBM patient data

From the PPI network analysis, we found that CDCP1 can directly affect CD44, FN1 and PTPRC and indirectly affect seven other genes to exert its functions (Fig. 8A). The PPI network was composed of 10 key genes and consisted of 11 nodes and 48 edges, with an average of 4.36 edges per node. We found that CDCP1 may directly affect the transformation of PN-GBM to MES-GBM by acting on CD44. To further study the possible mechanism by which CDCP1 promotes the transformation of PN-GBM to MES-GBM, we identified 10 key genes through the above strategies, among which some genes (CD44 [49], STAT3 [50], IL6 [51], and TLR2 [52]) have been reported to be related to the migration and invasion of GBM cells; therefore, CDCP1 is indeed related to the migration and invasion of GBM. On the other hand, these results suggest that other genes, such as ITGAM, may be related to invasion and migration. Among the 10 key genes, ITGAM, which is related to GBM immune infiltration, had the highest correlation with CDCP1, with a COR value of 0.668.

Based on the above results, we speculate that CDCP1 may promote the transformation of PN-GBM to MES-GBM by affecting the EMT process and immune infiltration of GBM and thus affect the prognosis of glioma patients. Thus, we generated an ROC curve based on the multivariate model of the interaction of CDCP1, CD44, and ITGAM. ROC curves were used to evaluate the predictive efficacy of CDCP1, CD44, ITGAM, and their combination in GBM patients. The areas under the curve (AUCs) for CDCP1, CD44, ITGAM and the markers combined were 0.558, 0.642, 0.528 and 0.692, respectively (Fig. 8B).

Based on the PPI network and correlation analyses of the key genes, three genes (CDCP1, CD44, and ITGAM) were integrated to establish a prognostic risk model. The risk scores from the prognostic risk model were calculated using the following formula: risk score = (1.25 * expression level of CDCP1) + (1.19 * expression level of CD44) + (1.09 * expression level of ITGAM). Glioma/GBM patients were divided into low-risk (n = 485/n = 187, respectively) and high-risk (n = 485/n = 187, respectively) groups according to the median risk score. The survival curve showed a poorer prognosis in the high-risk group than in the low-risk group (Fig. 8C-D). The prognostic risk model was verified with glioma patients from the TCGA database (Additional file 9: Fig. S9A–C). These findings show that our risk model can well indicate the prognosis of glioma/GBM patients.

### Combined CDCP1, CD44 and ITGAM expression can be used to predict the prognosis of glioma patients

To confirm the prognostic value of the risk signature, we constructed a nomogram based on the prognostic risk model, and we determined the clinical relevance and prognostic value of age, glioma type (primary glioma and recurrent glioma), sex, radiotherapy, TMZ chemotherapy, and IDH status. The 1-year, 3-year, and 5-year survival rates can be estimated from the total scores, which are the sum of the scores for each item, as shown in the nomogram (Fig. 8E–F). Analyses of the nomogram not only proved that the prognostic risk model is reliable but also showed that the accuracy of predicting survival in each patient was high. On the other hand, by comparing the factors in the nomograms, we found that the prognostic risk model had a high score, and this model played an important role.

## Discussion

Our study is the first to report that CDCP1 is a potential biomarker of the malignant phenotype of glioma and confirmed that the expression of CDCP1 increases with the grade of glioma. Based on these findings and the findings of the biological/functional analysis of CDCP1 in glioma, we hypothesize that CDCP1 can significantly promote the migration and invasion of glioma cells. To further analyze the potential molecular mechanism of CDCP1, we grouped patients in the CGGA database according to the median expression of CDCP1 and obtained 4007 differentially expressed genes. Then, we analyzed the correlation between these differentially expressed genes and CDCP1 and obtained 789 genes with high correlation. According to COR analysis, CDCP1 was highly expressed in MES-GBM and weakly expressed in PN-GBM. Subsequently, a PPI network of the 789 genes was obtained through the online website STRING, and 10 key genes (STAT3, PTPRC, FN1, IL1B, CXCL8, CD44, TLR2, IL10, IL6, and IFGAM) were identified through the MCODE and CytoNCA plug-ins of Cytoscape. We found that these 10 genes were positively correlated with CDCP1. Next, we established a prognostic risk model based on the expression of CDCP1, CD44 and ITGAM and verified the reliability and accuracy of our prognostic risk model by generating multivariate ROC curves and constructing a nomogram incorporating the diagnostic risk model and clinicopathological factors.

CD44 is a 99-kDa single-pass, transmembrane molecule that is very widely expressed in physiological and pathological contexts. Higher levels of CD44 make tumors more malignant, and patients with high levels of CD44 have short survival times [53–55]. Integrin alpha M (ITGAM, located on 16p11.2), also known as CD11b or complement receptor 3, which encodes the α-chain of the αMβ2 integrin, is an integrin adhesion molecule. CD11b + cells are the predominant infiltrating inflammatory cells in human gliomas [56].

A large number of studies have shown that CDCP1 affects patient prognosis by affecting the tumor. Recent oncology studies revealed that targeting CDCP1 reduced migration and tumor burden in high-grade serous ovarian cancer [57]. HJ Wright et al. indicated the therapeutic potential of targeting CDCP1 cleavage subtypes, as doing so inhibits triple-negative breast cancer metastasis [58]. Lijun et al. reported that the increased expression of CDCP1 promotes proliferation, migration, invasion, and EMT in cervical cancer [59]. However, the function and potential molecular mechanism of CDCP1 in glioma remain unclear.

We showed that CDCP1 plays an important role in glioma patients by examining data from the TCGA, CGGA and GEO databases. Then, bioinformatics analysis and experimental verification demonstrated that CDCP1's function is mainly related to EMT and immune infiltration, which are highly consistent with the characteristics of MES-GBM and the tumor microenvironment. COR analysis revealed that CDCP1 was highly expressed in MES-GBM and weakly expressed in PN-GBM. Therefore, we established a predictive risk model and verified the reliability of the model by performing immunohistochemistry and constructing a nomogram. Ultimately, we speculate that CDCP1, CD44 and ITGAM can be used to better diagnose glioma and predict the prognosis of glioma patients.

## Conclusions

In conclusion, we analyzed and verified that CDCP1 promotes the transformation of PN-GBM to MES-GBM by promoting the EMT process and immune infiltration, and we identified CD44 and ITGAM, which may interact with CDCP1, through a series of screening methods. By combining CDCP1, CD44 and ITGAM, a prognostic risk model was established and validated to predict 1-year, 3-year, and 5-year survival in glioma patients. The risk model was associated with glioma/GBM patient age, glioma type, sex, radiotherapy, TMZ chemotherapy, and IDH status. In summary, the risk model in our study can be used as a prognostic biomarker for gliomas.

## Supplementary Information

**Additional file 1: Figure S1.** The expression of CDCP1 according to the methylation status of the MGMT promoter (**A**), 1p/19q deletion status (**B**) and IDH mutation status (**C**) in the CGGA data.

**Additional file 2: Figure S2.** GSEA of GO functions (**A**) and KEGG pathways (**B**) of CDCP1.

**Additional file 3: Figure S3. **Correlation analysis between CDCP1 and the 20 upregulated and downregulated genes with the strongest correlations.

**Additional file 4: Figure S4.** GO functional analysis (**A**) and KEGG pathway analysis (**B**) of the 1923 downregulated genes.

**Additional file 5: Figure S5.** CDCP1 is highly expressed in MES-GBM according to TCGA data.

**Additional file 6: Figure S6.** Immune infiltration in GBM samples as assessed in CGGA data. The proportions of tumor-infiltrating immune cells in 22 GBM patients from the CGGA database (**A**). Correlation analysis between 22 kinds of tumor-infiltrating immune cells (**B**).

**Additional file 7: Figure S7.** Heatmap of 18 genes associated with CDCP1 expression.

**Additional file 8: Figure S8.** Differential analysis of the matrix score, immune score and ESTIMATE score in the CDCP1 high expression and low expression groups.

**Additional file 9: Figure S9.** Survival curve (**A**) and ROC curve (**B**) analyses of glioma patients based on the prognostic risk model and TCGA data. Nomogram (**C**) based on the risk model and clinicopathological factors.

**Additional file 10: Table S1.** Detailed information of nontumor and GBM patient samples from Nanfang Hospital.

## Data Availability

The datasets generated for this study can be found at https://cancergenome.nih.gov/, http://www.cgga.org.cn/, and https://www.ncbi.nlm.nih.gov/gds/?term=GSE50161.

## Abbreviations

CDCP1: CUB domain-containing protein 1
CNS: Central nervous system
GBM: Glioblastoma
MES: Mesenchymal
CL: Classical
NL: Neural
PN: Proneural
TCGA: The Cancer Genome Atlas
CGGA: Chinese Glioma Genome Atlas
GEO: Gene Expression Omnibus
ROC: Receiver operating characteristic
