# Supplementary figures and images for: Molecular mechanism by which CDCP1 promotes proneural-mesenchymal transformation in primary glioblastoma
Source: Cancer Cell Int. 2022 Apr 11;22:151. doi: 10.1186/s12935-021-02373-1 (PMC9003964; doi:10.1186/s12935-021-02373-1)

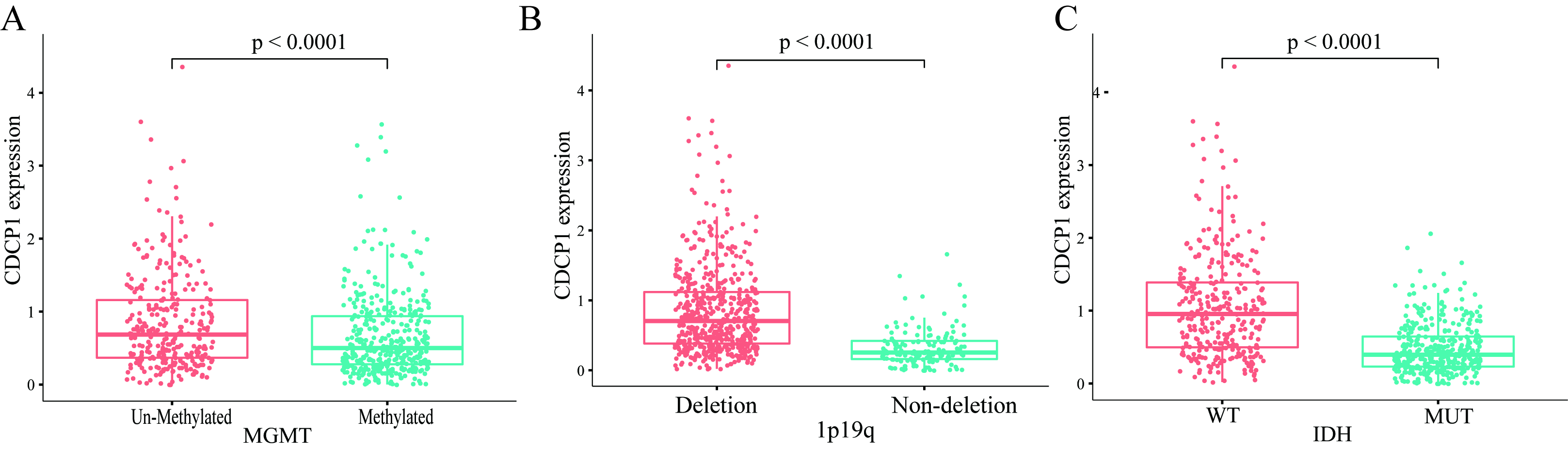

Supplement: Supplementary file 1 — Additional file 1: Figure S1. The expression of CDCP1 according to the methylation status of the MGMT promoter (A), 1p/19q deletion status (B) and IDH mutation status (C) in the CGGA data. [file 12935_2021_2373_MOESM1_ESM.tif]

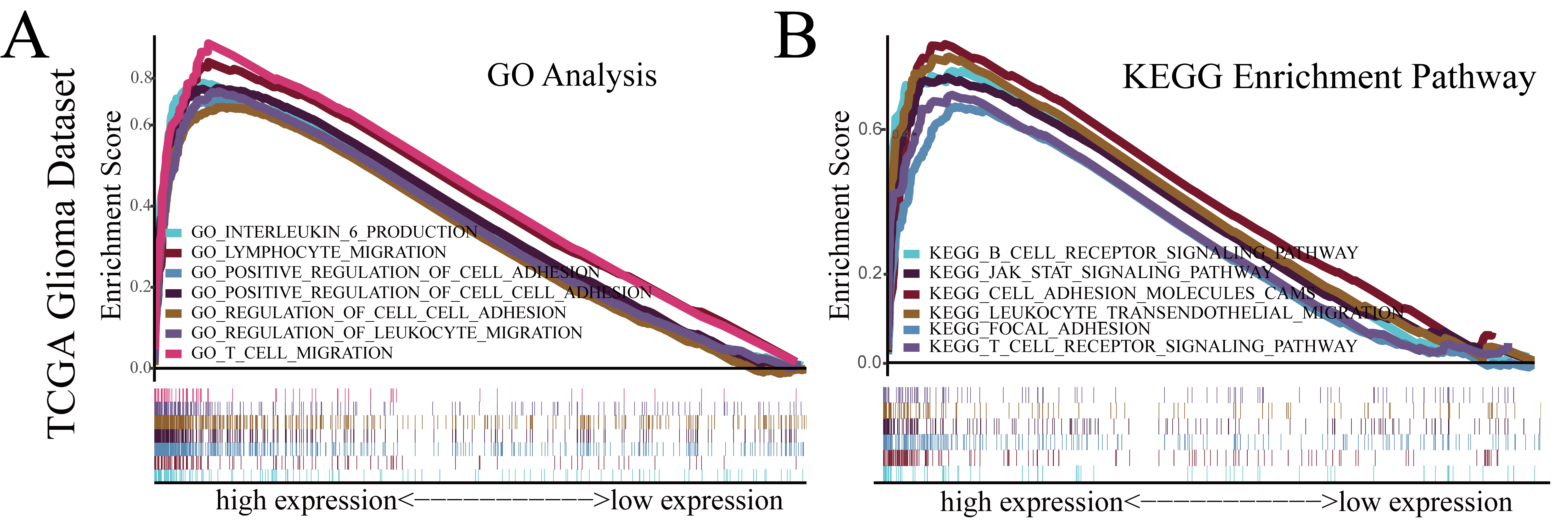

Supplement: Supplementary file 2 — Additional file 2: Figure S2. GSEA of GO functions (A) and KEGG pathways (B) of CDCP1. [file 12935_2021_2373_MOESM2_ESM.tif]

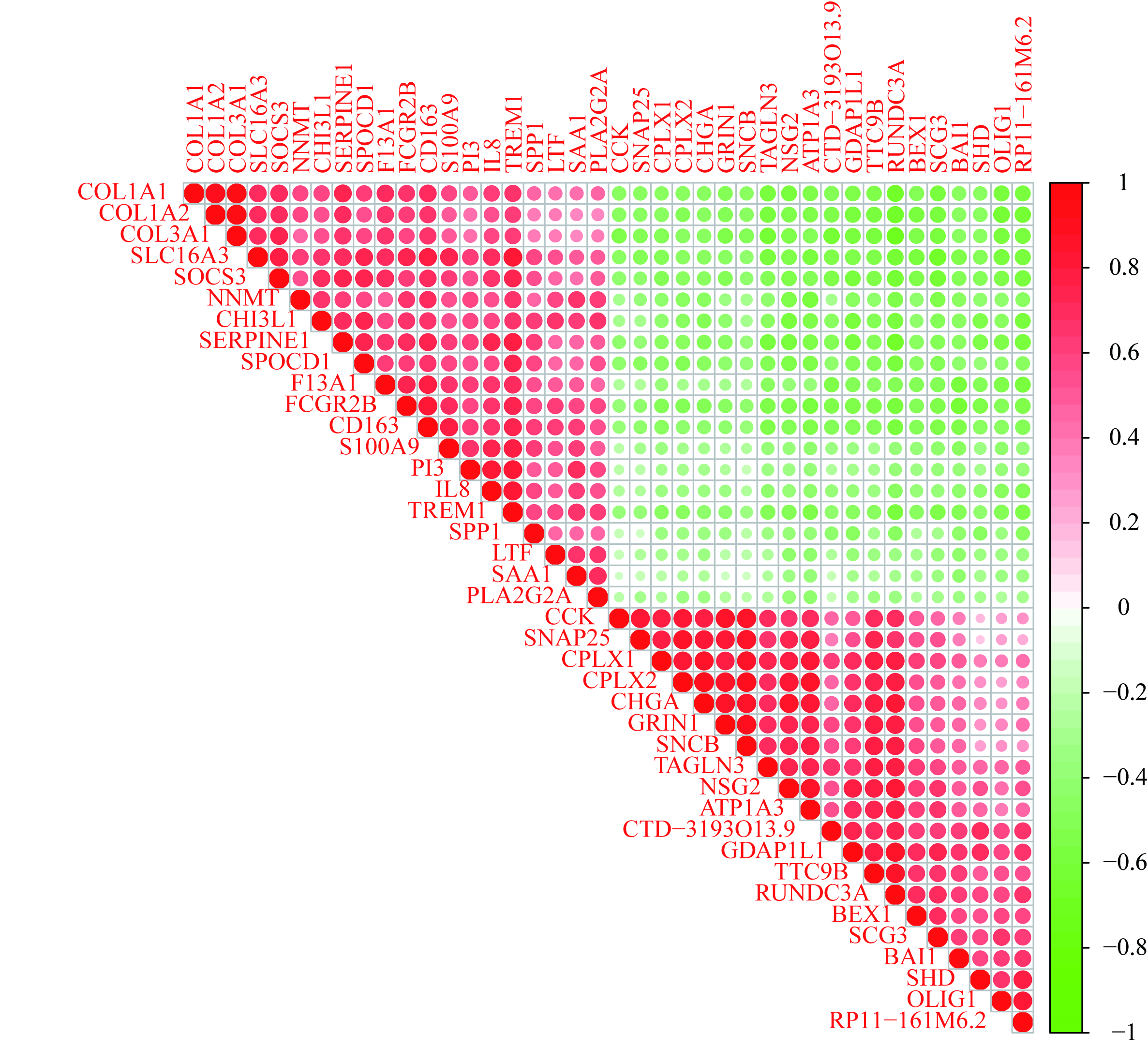

Supplement: Supplementary file 3 — Additional file 3: Figure S3. Correlation analysis between CDCP1 and the 20 upregulated and downregulated genes with the strongest correlations. [file 12935_2021_2373_MOESM3_ESM.tif]

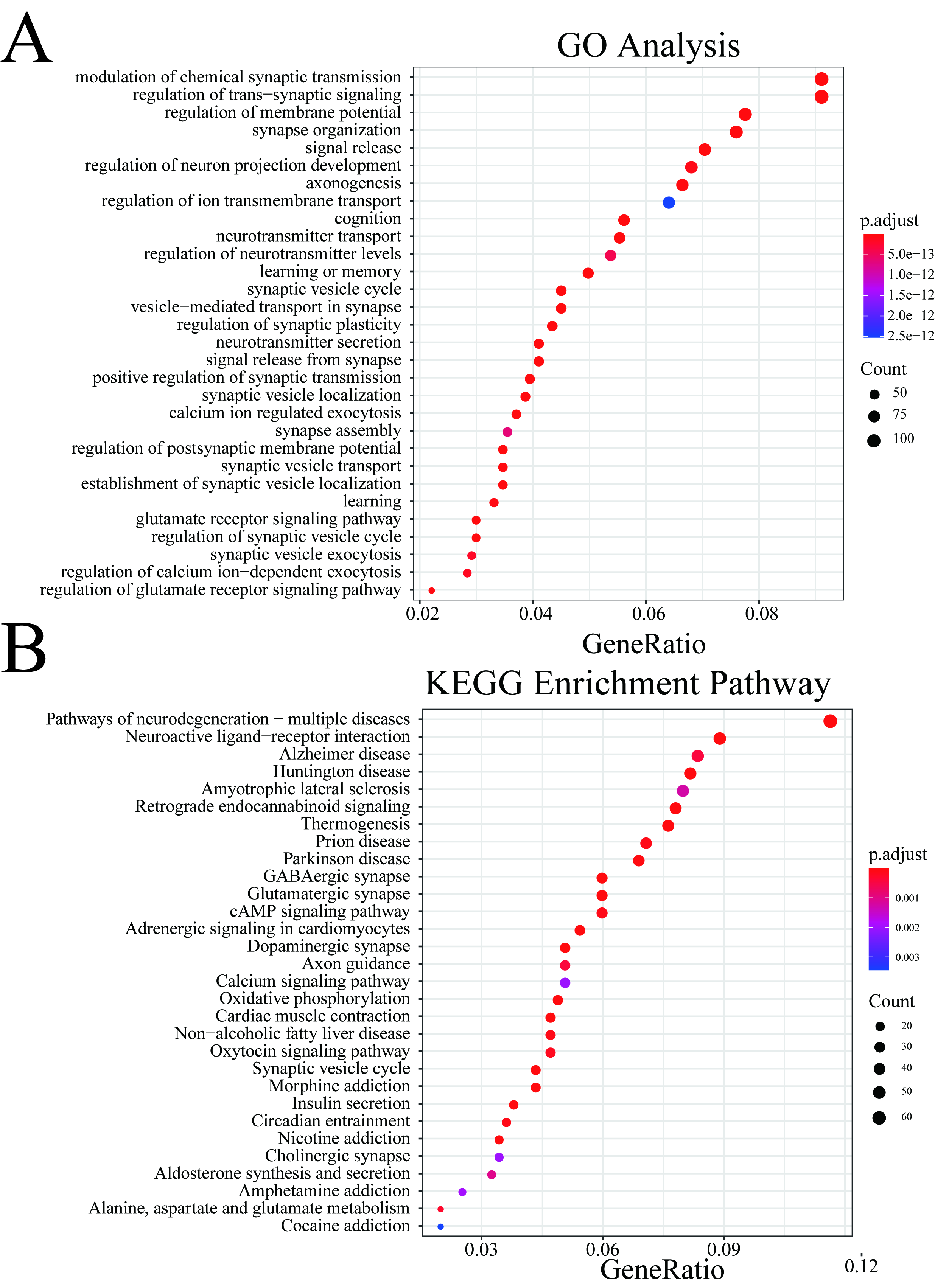

Supplement: Supplementary file 4 — Additional file 4: Figure S4. GO functional analysis (A) and KEGG pathway analysis (B) of the 1923 downregulated genes. [file 12935_2021_2373_MOESM4_ESM.tif]

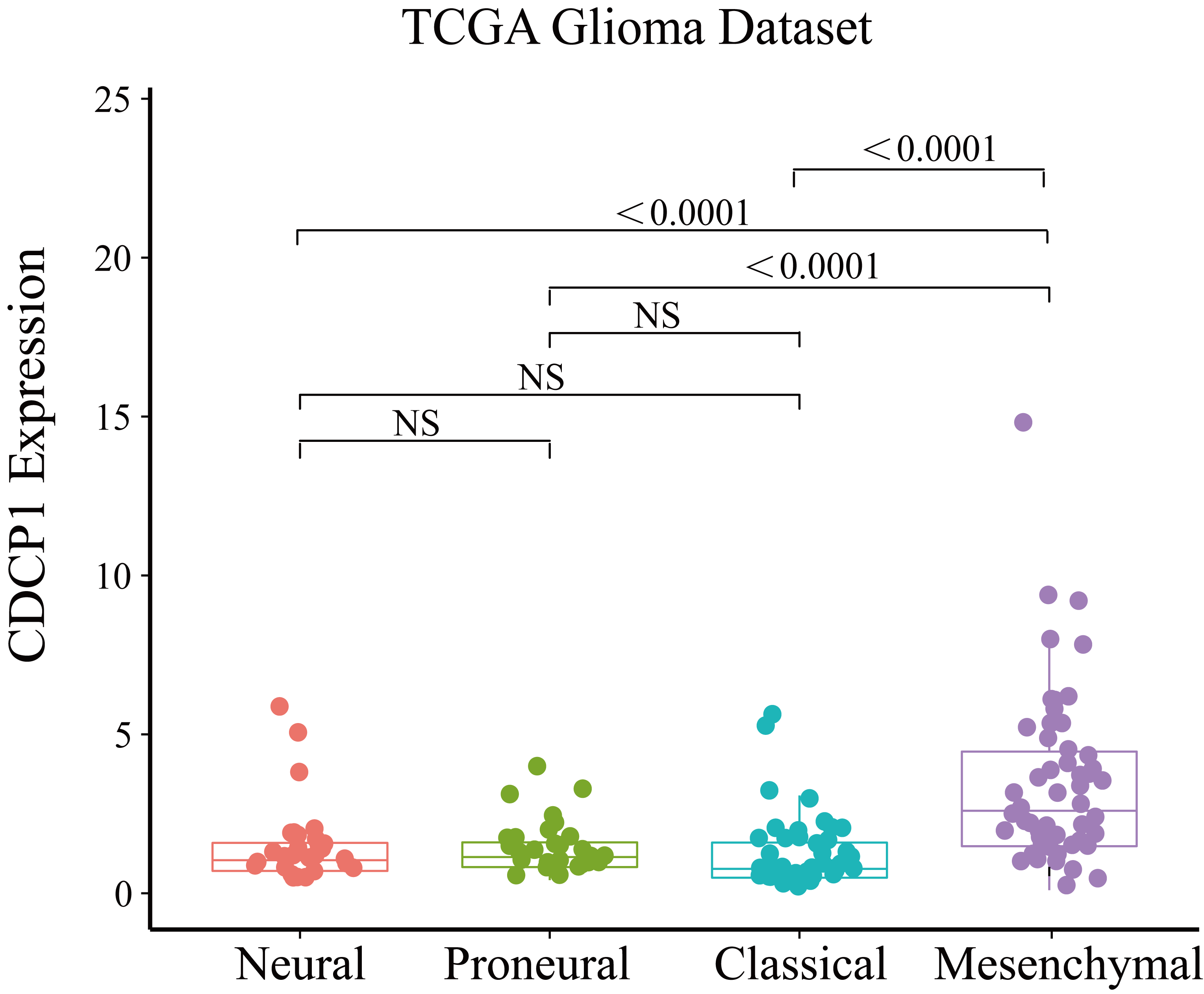

Supplement: Supplementary file 5 — Additional file 5: Figure S5. CDCP1 is highly expressed in MES-GBM according to TCGA data. [file 12935_2021_2373_MOESM5_ESM.tif]

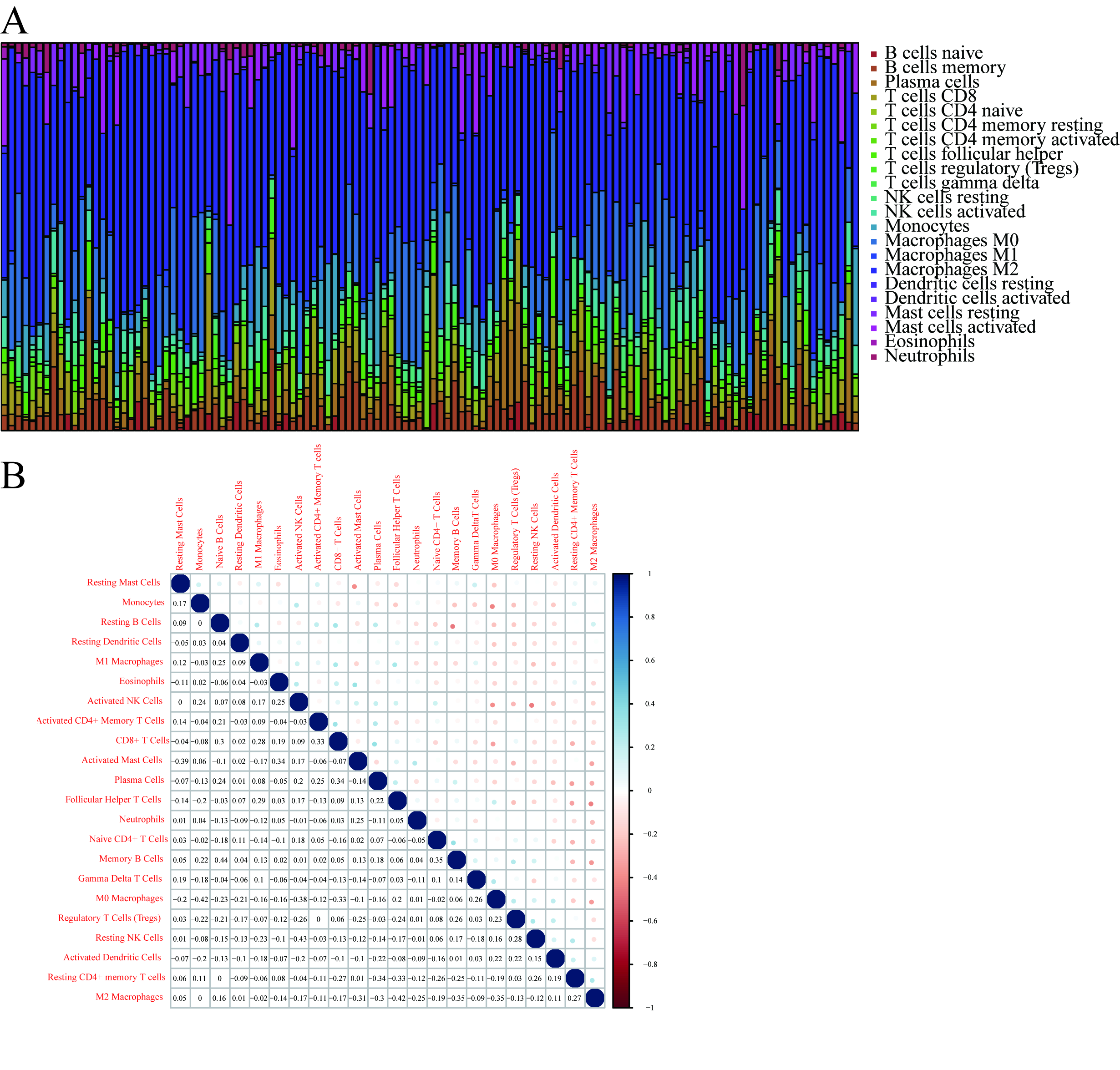

Supplement: Supplementary file 6 — Additional file 6: Figure S6. Immune infiltration in GBM samples as assessed in CGGA data. The proportions of tumor-infiltrating immune cells in 22 GBM patients from the CGGA database (A). Correlation analysis between 22 kinds of tumor-infiltrating immune cells (B). [file 12935_2021_2373_MOESM6_ESM.tif]

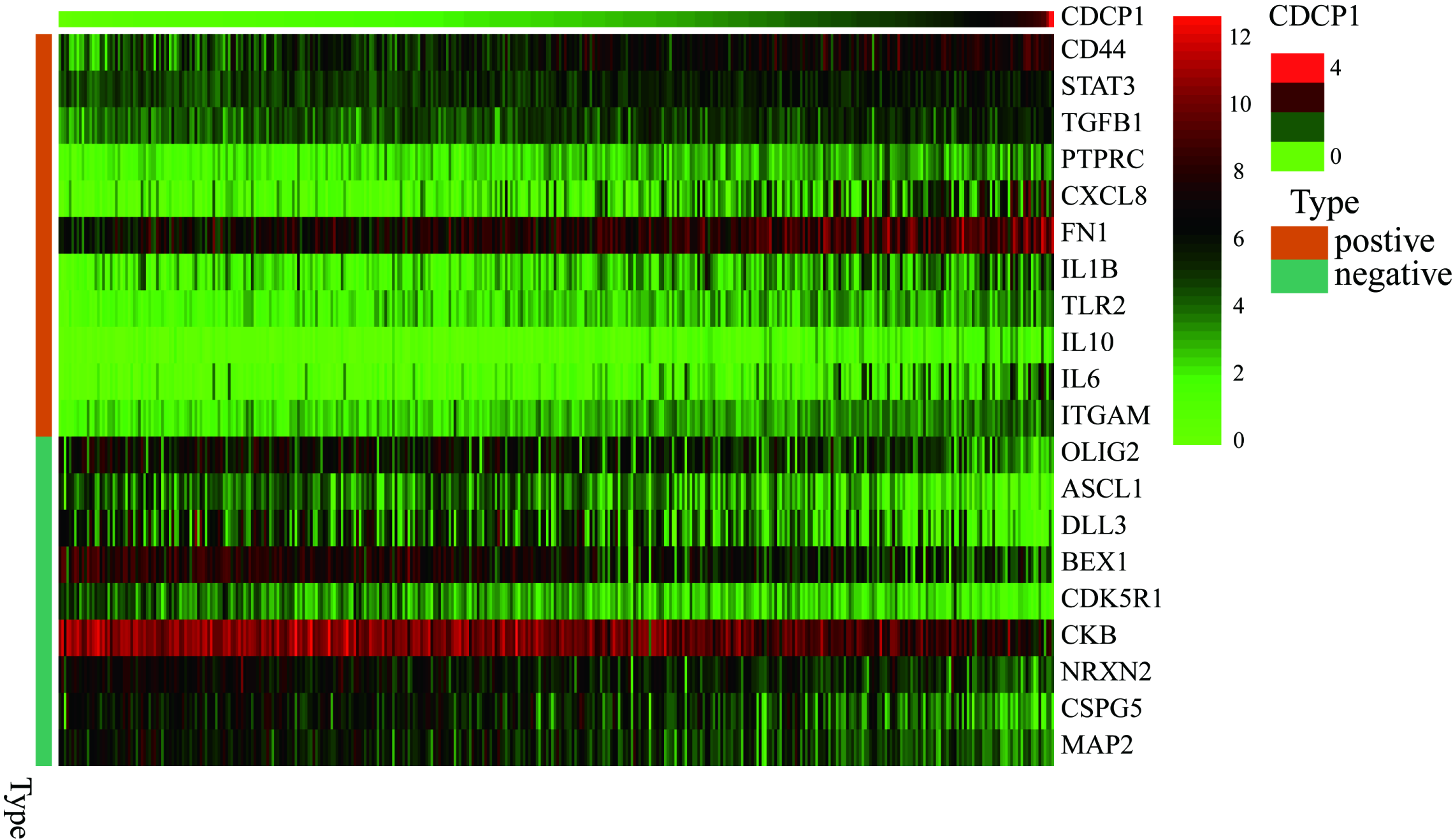

Supplement: Supplementary file 7 — Additional file 7: Figure S7. Heatmap of 18 genes associated with CDCP1 expression. [file 12935_2021_2373_MOESM7_ESM.tif]

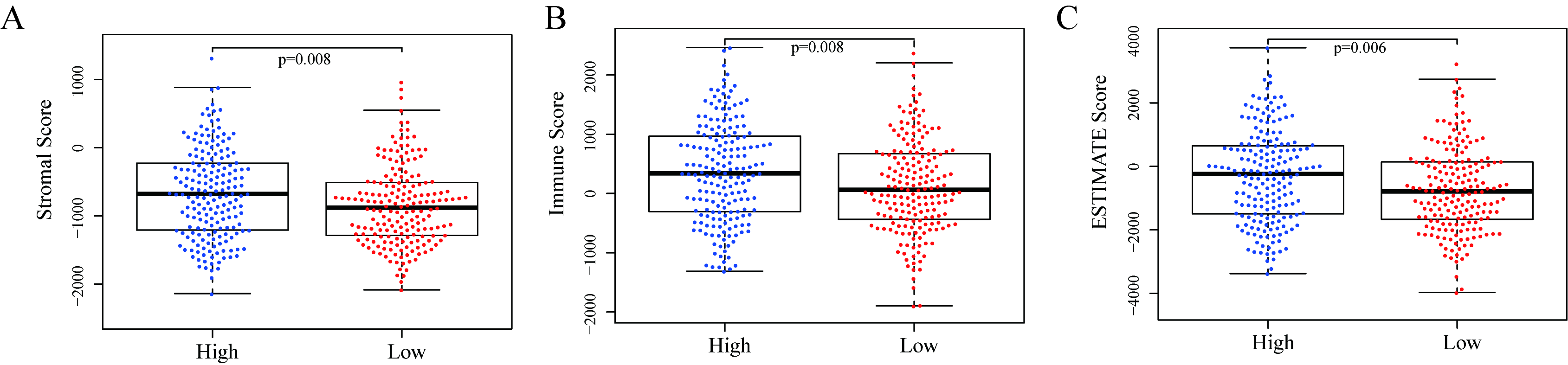

Supplement: Supplementary file 8 — Additional file 8: Figure S8. Differential analysis of the matrix score, immune score and ESTIMATE score in the CDCP1 high expression and low expression groups. [file 12935_2021_2373_MOESM8_ESM.tif]

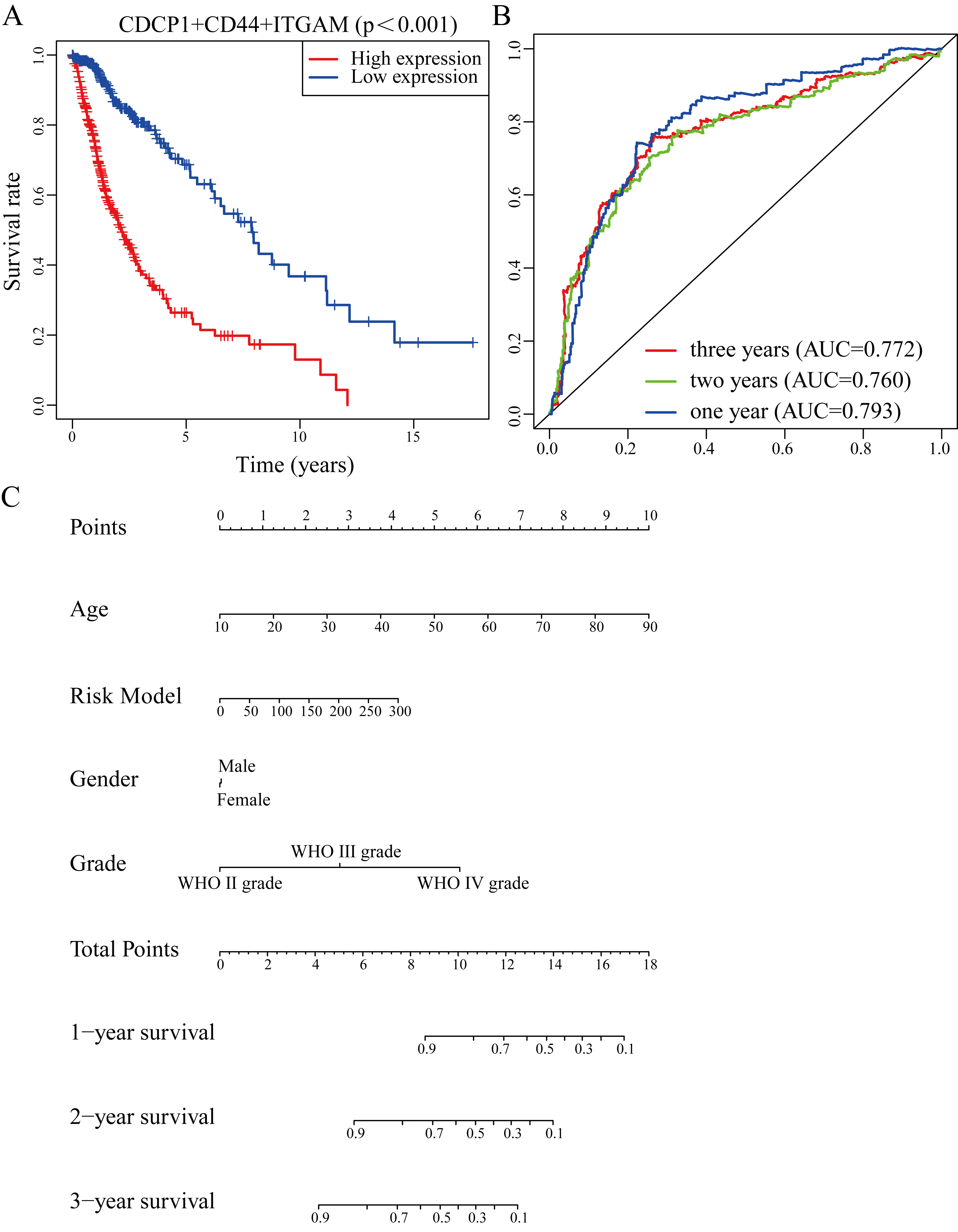

Supplement: Supplementary file 9 — Additional file 9: Figure S9. Survival curve (A) and ROC curve (B) analyses of glioma patients based on the prognostic risk model and TCGA data. Nomogram (C) based on the risk model and clinicopathological factors. [file 12935_2021_2373_MOESM9_ESM.tif]
